# Acute Reperfusion Therapies for Acute Ischemic Stroke

**DOI:** 10.3390/jcm10163677

**Published:** 2021-08-19

**Authors:** Rajeel Imran, Ghada A Mohamed, Fadi Nahab

**Affiliations:** Department of Neurology, Emory University, Atlanta, GA 30322, USA; rimran2@emory.edu (R.I.); gamoham@emory.edu (G.A.M.)

**Keywords:** ischemic stroke, thrombolysis, thrombectomy

## Abstract

The field of acute stroke treatment has made tremendous progress in reducing the overall burden of disability. Understanding the pathophysiology of acute ischemic injury, neuroimaging to quantify the extent of penumbra and infarction, and acute stroke reperfusion therapies have together contributed to these advancements. In this review we highlight advancements in reperfusion therapies for acute ischemic stroke.

## 1. Introduction

Acute ischemic stroke continues to pose a significant challenge for our healthcare system. Approximately 795,000 people suffer a new or recurrent stroke each year just in the United States with current projections showing an additional 3.4 million adults having a new stroke by the year 2030 [1]. Approximately 85% of strokes are caused by an ischemic event and among those who survive a significant number require assistance with their daily activities [1].

Despite these grim numbers, there have been several notable advances in the past two decades that have transformed our approach to both diagnosis and treatment of patients with acute ischemic stroke. The widespread use of intravenous alteplase and now endovascular therapy has led to significant improvement in functional outcomes with notable reductions in the rates of disability. More recently, the focus has shifted to extending these therapies beyond the previously defined “time-windows”. Indeed, there is growing evidence suggesting the need to re-define and personalize these therapeutic strategies based on several key factors including advanced neuro-imaging characteristics.

The purpose of this review is to summarize the principles and current understanding of the treatment options available for ischemic stroke focused on the hyperacute and acute stages.

## 2. Basic Principles of Acute Stroke Reperfusion Therapy

Acute ischemic stroke occurs due to sudden obstruction of blood flow of a cerebral artery leading to lack of oxygenation of the downstream brain tissue. If the obstruction persists for a considerable time, it can result in permanent loss of neurological function mediated by the affected region of the brain. Pre-clinical studies have estimated that approximately 1.9 million neurons can undergo cell-death following each minute of ischemia [2]. However, if the blockage is removed before substantial tissue injury occurs, reperfusion of the ischemic tissue may reverse such neuronal damage. 

Thus, an important component of acute reperfusion therapies for acute ischemic stroke involves the concept of the infarct core, the region of the brain which has suffered irreversible damage, versus ischemic penumbra, defined as the critically hypo-perfused area of brain that is potentially salvageable but is progressing to complete infarction in the absence of timely reperfusion. 

This time duration of ischemic tissue viability varies considerably and depends on several factors such as the location of cerebral artery occlusion, extent of the occlusion (partial versus complete), degree of cerebral collateral blood flow, and cerebral perfusion pressure. In particular, the extent of recruitment of cerebral collaterals has been demonstrated to be one of the strongest predictors of favorable outcome in ischemic stroke patients [3]. Several studies have suggested favorable collateral flow with slowed progression of ischemic penumbra to infarct core and better clinical outcomes following acute interventions [4,5,6]. Understanding their role in cases of acute ischemic stokes has also been pivotal in garnering utility of acute reperfusion therapeutics beyond the previously recognized time windows. 

Moreover, in recent years, the use of advanced neuro-imaging modalities such as CT perfusion or magnetic resonance (MR) diffusion/perfusion have become more prevalent especially at tertiary care hospitals. These imaging techniques especially when used alongside CT or MR angiography provide not only an assessment of the collateral circulation but also the ability to visualize and differentiate potential ischemic tissue at risk from the irreversibly damaged infarct core. CT-based perfusion maps, the more widely adopted imaging modality at the present time, can estimate the region of ischemic penumbra by computing the delay in arrival of contrast to the affected brain tissue (mean transit time). At the same time, thresholds of cerebral blood volume or cerebral blood flow below critical values have been shown to predict regions of completed infarction. Several automated volumetric software tools such as RAPID AI (iSchema View, Inc., Menlo Park, CA, USA), Viz.AI© (San Francisco, CA, USA), and Brainomix© (Oxford, UK) have been developed based on computational algorithms and machine learning. These software can in turn rapidly provide estimates for infarct core and penumbra on the basis of pre-defined empirical threshold values. 

MR-based diffusion-weighted imaging (DWI) has also been utilized in the hyperacute stage of acute ischemic stroke management at select stroke centers. Some comparative studies have demonstrated superiority of MR-DWI protocols over CT perfusion maps for prediction of ischemic core [7], especially in cases of subcortical lesions [8,9,10], though its practical utility in the “real-world” setting remains relatively limited due to prolonged acquisition times compared to CT as well as 24/7 availability. In addition, MR perfusion protocols have been developed, which provide an alternative and arguably more reliable assessment of the penumbral tissue [7,11,12], especially in the later time windows, when compared to CT perfusion.

## 3. Acute Reperfusion Treatment Options

### 3.1. Intravenous Thrombolysis

Intravenous thrombolysis has remained the mainstay of reperfusion therapy for acute ischemic stroke patients since the 1990s. However, there have been some significant advancements that have expanded the eligibility criteria for patients to receive these therapies over an expanded time window. In addition, newer thrombolytic agents such as Tenecteplase provide the promise of better safety outcomes and lower cost of drug administration.

#### 3.1.1. Alteplase 

Alteplase or recombinant tissue plasminogen activator (tPA) has been the first-line therapy for reperfusion in acute ischemic stroke patients since its initial approval following the landmark NINDS trial, published in 1995 [13]. Alteplase bears affinity to the fibrin component of a blood clot and sets off a fibrinolytic cascade by converting plasminogen to plasmin, leading to the dissolution of fibrin. Intravenous (IV) alteplase has shown benefit for patients with disabling stroke regardless of the NIHSS score, though it is not clearly recommended for those with nondisabling strokes with a low NIHSS score [13]. Other exclusion criteria for IV alteplase administration from the American Heart Association (AHA)/American Stroke Association (ASA) stroke guidelines can be found in Table 1. 

The original NINDS trial treated patients up to three hours from the time of initial stroke symptom onset. The study confirmed that patients who received IV alteplase as compared to placebo were at least 30% more likely to have favorable clinical outcomes at 90-days (odds ratio (OR) 1.7; *p* = 0.008). This benefit of treatment was present despite an increased risk of symptomatic intracerebral hemorrhage (ICH) of 6.4% with IV alteplase; however, no significant difference in 90-day mortality was noted (17% vs. 21%; *p* = 0.30) [13]. 

A subsequent patient level data meta-analysis from 2004 included all previous studies involving IV alteplase for acute ischemic stroke and showed that favorable outcomes extended up to 4.5 h from stroke symptom onset [14]. The results of this analysis became the basis for the ECASS III trial, which randomized 821 patients to receive either intravenous alteplase or placebo within 3 to 4.5 h from stroke onset [15]. These results confirmed favorable 90-day clinical outcomes in the drug therapy group compared to placebo, and thus led to extension of the recommended treatment time window from 3 to 4.5 h as long as patients met the trial’s enrollment criteria. It is however important to note that the benefit in this added time window was less robust with a number needed to treat (to achieve favorable functional outcomes) of 14 as compared to 7 within the first 3 h of stroke onset from the NINDS study. A pooled data analysis from 2009 also showed similar findings supporting the continued need for earlier administration of thrombolytic therapy [16]. A pre-specified meta-analysis of individual patient data from nine randomized trials consisting of 6756 patients showed not only the continued need for earlier administration of thrombolytic therapy but also that the efficacy of alteplase was present irrespective of age or stroke severity [17].

These pivotal trials also became the basis for eligibility of patients to receive the thrombolytic therapy along with the mode and dose of medication administration. The alteplase dose is calculated according to the patient’s body weight (0.9 mg/kg), with a maximum dose of 90 mg, of which 10% is given as a loading bolus over 1 min and the remainder administered through an intravenous infusion over 1 h. Notably, there are some regions including Japan where a smaller dose of 0.6 mg/kg is utilized owing to concerns for increased complications and bleeding risks in their specific patient populations [18,19]. These regional recommendations have been supported by the results of the ENCHANTED study (Enhanced Control of Hypertension and Thrombolysis Stroke Study) that enrolled 3310 predominantly Asian patients to receive either the standard 0.9 mg/kg or a lower 0.6 mg/kg of IV alteplase within 4.5 h of stroke onset [20]. Although the study failed to show non-inferiority of the lower alteplase dose for the end points of death or disability, there was a statistically lower risk of symptomatic ICH in the lower dose arm.

As previously discussed, one important consideration in terms of eligibility of IV thrombolytic therapy is time from last known well (LKW). There is data available to suggest that as much as 25 to 30% of patients with acute ischemic stroke have an unwitnessed symptom onset, previously excluding them from potential acute therapy [21,22]. This includes a significant proportion of patients who are noted to have stroke symptoms upon awakening from sleep or are aphasic and thus unable to report a clear time of stroke onset. Over the past several years, there has been growing evidence suggesting that carefully selected patients from these subgroups of either “wake-up” strokes or strokes with unknown time of onset may still benefit from acute thrombolytic therapies. This remains a possibility as there were studies that suggested that in cases of wake-up stroke, the actual onset of symptoms occurs in the few hours immediately before awakening [23]. In addition, a subset of patients with unclear time of stroke onset may still be within the 4.5-h time window that otherwise simply could not be determined. 

Simultaneously, advances in MR neuroimaging led to the conclusion that possible imaging biomarkers may be available to ascertain whether the stroke symptom onset was within 4.5 h of the imaging time. One such promising avenue was the imaging signal mismatch between MRI DWI and fluid-attenuated inversion recovery (FLAIR) sequences, with the DWI-positive and FLAIR-negative pattern suggesting a shorter duration from symptom onset [24,25,26]. This DWI-FLAIR imaging mismatch was used to identify patients with unclear time of onset in the randomized, controlled WAKE-UP trial, which compared IV alteplase to placebo; 503 patients were enrolled, of which 94% had stroke symptoms upon awakening [27]. The results showed a significant benefit of IV thrombolysis on excellent functional outcomes at 90-days when compared to placebo (53.3% versus 41.8% respectively; adjusted OR, 1.61; 95% CI, 1.09 to 2.36; *p* = 0.02). There was however a non-statistically significant trend towards increased risk of symptomatic ICH in the treatment arm compared to placebo (2% versus 0.4% respectively) and death (4.1% versus 1.2% respectively). These results were supported by the EXTEND Trial, which randomized patients to receive IV thrombolysis with alteplase or placebo between 4.5 to 9 h from LKW using advanced MR and CT perfusion imaging for patient selection [28]. Though the number of patients included was small, and the benefit less robust, the treatment arm did result in a higher percentage of patients with no to minor neurologic deficits than with placebo. Two subsequent pooled analyses published in 2019 and 2020 confirmed this treatment benefit, though there were some notable differences in the imaging selection modalities utilized in the individual studies [29,30]. This benefit remained present despite considering the increases in ICH rates from the pooled data. 

Following the publication of these trials, in 2019 the AHA/ASA stroke guidelines were updated and suggested the use of IV alteplase based on DWI-FLAIR mismatch or CT perfusion to select patients beyond the 4.5-h time window who have an unclear time of stroke onset or awoke with stroke symptoms [31]. 

#### 3.1.2. Tenecteplase

Tenecteplase is a genetically modified variant of alteplase with greater fibrin specificity, increased resistance against tissue plasminogen activator inhibitor-1 and a longer plasma half-life than alteplase, thus permitting a single-dose intravenous bolus administration. More recently, there has been tremendous interest in its utility for the treatment of acute ischemic stroke especially after reports of its comparable efficacy and overall lower risks of bleeding compared to alteplase in a trial of patients with ST-segment elevation myocardial infarction [32]. NOR-TEST (Norwegian Tenecteplase Stroke Trial) was a randomized, phase III, controlled trial that assessed the efficacy and safety of tenecteplase 0.4 mg/kg (up to 40 mg) versus the standard-dose of alteplase within 4.5 h of acute ischemic stroke onset; 1100 patients were included with the majority having mild stroke symptoms (median NIHSS score of 4) [33]. The results showed similar efficacy between both agents in terms of excellent functional outcome and safety profile at 90 days. 

Subsequently, the results of the EXTEND-IA TNK (Tenecteplase Versus Alteplase Before Endovascular Therapy for Ischemic Stroke) trial were published and included 202 patients with acute ischemic stroke and a concurrent proximal intracranial artery occlusion (internal carotid artery (ICA), middle cerebral artery (MCA) or basilar artery) [34]. These mechanical thrombectomy eligible patients were randomized to receive either 0.25 mg/kg of tenecteplase (up to 25 mg) or standard-dose of alteplase within 4.5 h of stroke symptom onset. The primary outcome was determined based on the degree of reperfusion/recanalization rates as noted on conventional cerebral angiogram prior to undergoing mechanical thrombectomy. Interestingly, patients treated with tenecteplase had significantly higher rates of the primary outcome compared to alteplase (22% versus 10% respectively) and better functional outcomes at 90 days. The rates of symptomatic ICH were low and comparable in both groups (1%). 

Since then, a meta-analysis consisting of five randomized trials comparing tenecteplase to alteplase has been published [35]. The analysis included a total of 1585 patients with the results showing non-inferiority of tenecteplase versus alteplase for the treatment of acute ischemic stroke in terms of excellent functional outcome (58% versus 56%; OR 1.17; 95% CI 0.95–1.44; *p* = 0.13). Rates of symptomatic ICH were again similar in both groups (3%). It is important to note that there were considerable differences in the dosing amount in each of the trials and the optimal dose for tenecteplase at this time remains unclear. 

Based on the aforementioned trials, the 2019 AHA/ASA updated its recommendations to suggest consideration of using tenecteplase (single IV bolus of 0.25 mg/kg, with maximum dose of 25 mg) over alteplase in patients with acute ischemic stroke who are also eligible to undergo mechanical thrombectomy.

### 3.2. Endovascular Mechanical Thrombectomy

It has widely been observed that patients with an acute ischemic stroke secondary to a proximal intracranial large vessel occlusion (LVO) benefit significantly less from IV alteplase. One proposed hypothesis to explain this finding has been lower levels of plasminogen near the center of these larger volume blood clots, thus providing less surface area for agents such as alteplase to assert their enzymatic function leading to poor recanalization rates [36]. Indeed, data from several observational studies have noted successful recanalization rates in cases of proximal LVOs treated with alteplase to be less than 20%, correlating with poor clinical outcomes [37,38,39,40,41,42].

Up until 2013, several clinical trials comparing endovascular treatments to the standard of care (including alteplase) for acute ischemic stroke with a LVO failed to show clear benefit of endovascular therapy [43,44,45]. Several factors may have contributed to the overall disappointing results including patient selection, trial designs and the nature of first-generation devices and aspiration catheters. 

However, development of stent retriever devices enhanced clot extraction capabilities and led them to be FDA-approved for use in six subsequent randomized clinical trials [46,47,48,49,50,51]. These trials primarily enrolled adult acute ischemic stroke patients who had an LVO in the intracranial internal carotid artery (ICA) or the first segment of the middle cerebral artery (M1). In addition, the selection criteria were refined to include mostly those individuals who had severe neurological deficits at the time of presentation with minimal pre-stroke disability. The main features of these randomized controlled trials are summarized in Table 2.

The results of these trials showed a dramatic improvement in functional outcomes in patients with acute stroke due to LVOs, with the numbers needed to treat to prevent significant disability or death ranging from 3 to 7. The first of these published trials, MR CLEAN included 500 patients with acute ischemic stroke and a proximal LVO less than six hours from LKW across 16 medical centers in the Netherlands [45]. Results showed an absolute increase of 13.5% (95% CI 5.9–21.2) in the rate of functional independence in favor of endovascular treatment (32.6% versus 19.1%). Owing to the tremendous potential for benefit in the endovascular arm, following the publication of the MR CLEAN trial, an interim analysis of the other four ongoing trials (ESCAPE, EXTEND-IA, SWIFT PRIME, and REVASCAT) was done, which confirmed the significant benefit of mechanical thrombectomy. In 2015, a meta-analysis of pooled patient-level data from these five randomized trials confirmed that the number needed to treat with mechanical thrombectomy to reduce risk of disability by at least one level on the modified Rankin Scale (mRS) score for one patient was 2.6 [52]. The benefit was noted across multiple subgroups, including patients older than 80 years, those with a baseline NIHSS score of more than 20 (severe stroke) and amongst patients who were otherwise ineligible for IV alteplase use. Moreover, mechanical thrombectomy was considered to be safe with a combined rate of symptomatic ICH of 4.4%. Soon thereafter, based on the selection criteria and results of these pivotal trials, the AHA/ASA stroke guidelines were updated in 2015 to include mechanical thrombectomy treatment with stent-retrievers recommended in the first 6 h of acute stroke onset if the pre-requisites of inclusion were fulfilled. 

In 2018, publication of the DAWN and DEFUSE 3 trials demonstrated benefit of mechanical thrombectomy performed beyond 6 h and up to 24 h after the onset of acute stroke symptoms and expanded the treatment window for patients with LVO [53,54]. The patient selection was based on a mismatch between the ischemic core and penumbra displayed in perfusion images. The aim of reperfusion therapy is to prevent the expansion of this infarct core by salvaging the region of ischemic penumbra. The use of neuroimaging to identify brain tissue viability could therefore identify a subset of patients who could benefit from mechanical thrombectomy beyond standard treatment time windows. 

The DWI or CTP Assessment with Clinical Mismatch in the Triage of Wake Up and Late Presenting Strokes Undergoing Neurointervention with Trevo (DAWN) trial was a multi-center, randomized controlled trial, which enrolled patients between 6 to 24 h from onset of stroke symptoms who had a proximal LVO [53]. The inclusion of patients was based on a mismatch between the extent of ischemic core as identified by pre-defined DWI or CT perfusion parameters and severity of neurological deficits (defined as NIHSS score of 10 or more). In the study, the median time from onset of stroke symptoms to intervention was 12.5 h. The results demonstrated a clear and dramatic increase in patients with minimal or no disability undergoing mechanical thrombectomy compared to standard therapy (49% versus 13% respectively; adjusted difference, 33%; 95% CI, 21 to 44). 

**Figure 1:**
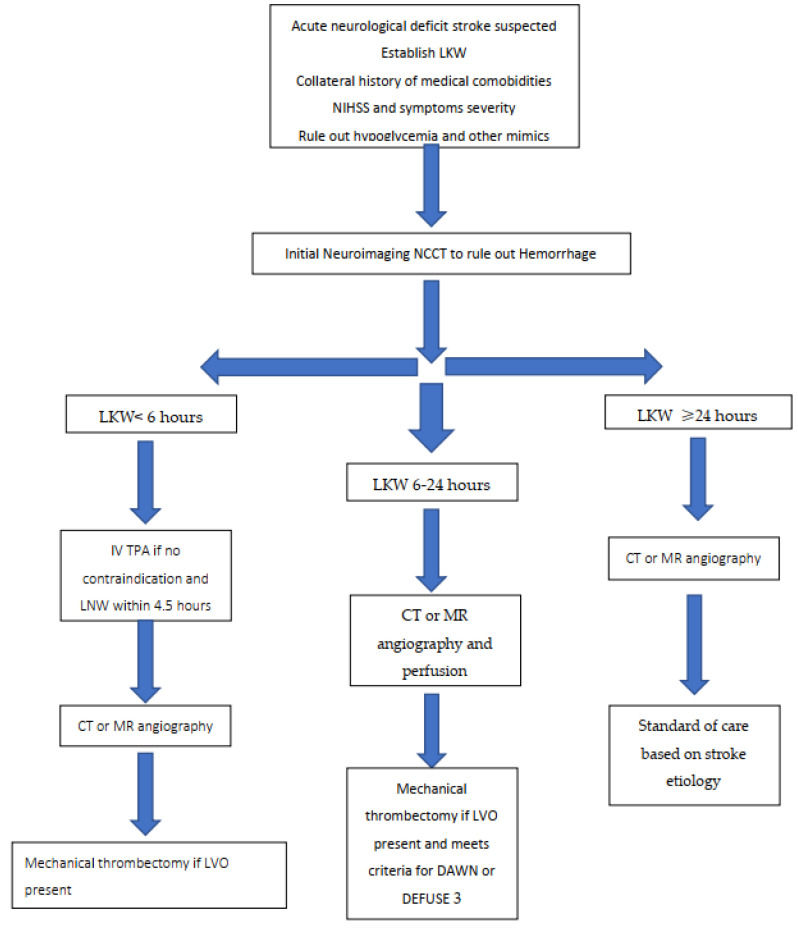
Stepwise Algorithm for Initial Management of Acute Ischemic Stroke in Adults.

Similarly, the Endovascular Therapy Following Imaging Evaluation for Ischemic Stroke (DEFUSE) 3 trial was a multi-center, randomized trial enrolling acute ischemic stroke patients with a proximal LVO 6 to 16 h from LKW [54]. Median time to treatment with mechanical thrombectomy in the study was 11 h from LKW. The DEFUSE 3 trial included patients with less severe disability on presentation (NIHSS of 6 or more) and allowed pretreatment core infarct volumes of up to 70 mL as long as they had substantially more ischemic penumbral volumes (based on CT or MR perfusion imaging). The results again demonstrated a significantly higher percentage of patients who achieved functional independence at 90-days in the intervention arm as compared to standard management (45% versus 17% respectively, relative risk, 2.67; 95% CI, 1.60 to 4.48, *p* < 0.001). Notably, there was an additional 20% absolute reduction in risk of death or severe disability in favor of mechanical thrombectomy. The intervention arms in both studies were also deemed safe with no significant increase in the rate of symptomatic ICH as compared to the control arms. These benefits of endovascular therapy for this extended window was maintained across all observed subgroups, including age and mode of presentation. The AHA/ASA stroke guidelines were updated in 2018 with recommendations to offer mechanical thrombectomy up to 24 h from LKN in patients who meet trial inclusion and exclusion criteria [31]. Based on current guidelines, a summary of the steps involved in the hyperacute to acute management of acute ischemic stroke patients with and without a LVO can be found in Figure 1.

#### Special Considerations in Thrombectomy Treatment and Combination Therapy

Intravenous thrombolysis has been a standard of care for any patient presenting with acute ischemic stroke within 4.5 h, regardless of the occlusion site. Recent studies have addressed the effectiveness of IV TPA in patients presenting with LVO especially if they have proximal occlusion or high clot burden. The primary challenge is that IV alteplase prior to MT has been reported to have low recanalization rates of proximal occlusion, with frequencies as low as 4% for ICA and 7% for proximal MCA. Further, there is an increased risk of clot migration, distal migration that hampers MT recanalization scores, and an increased risk of ICH. On the other hand, IV alteplase has shown effectiveness in distal MCA occlusion as well as facilitation of MT with thrombolysis of clot fragments during stent retrieval [55]. Recent randomized trials have improved our understanding of the optimal treatment algorithm.

The Direct-MT trial compared direct MT to a combination of IV alteplase with MT. It failed to show non inferiority of direct MT over MT+alteplase for achieving good clinical outcome [56]. The SKIP trial also compared thrombectomy with or without IV alteplase in Japan and failed to demonstrate any significant difference between both groups. The EXTEND-IA TNK trial utilized Tenecteplase prior to MT to establish an effective dose and showed that 0.25 mg/kg does not show any significant difference in rates of recanalization or functional outcomes compared to 0.4 mg/kg [57]. Prospective randomized trials are currently enrolling patients to determine the role of IV thrombolysis prior to MT.

## 4. Future Directions

Ongoing research aims to optimize treatment protocols and to identify additional patient subgroups who may benefit from acute reperfusion therapy. Early pre-hospital recognition of stroke signs and appropriate triage to stroke centers also remain critical to reductions in treatment times and disability. Innovative strategies in community education, pre-hospital scales to guide emergency medical services to the appropriate stroke treatment center and mobile stroke units with embedded CT scanners will improve our goal of getting the right patients to the right treatment centers at the right time. Ongoing efforts are refining our patient selection to identify LVO patients who may not benefit from “bridging” treatment with IV thrombolytic therapy prior to thrombectomy versus a direct-to-thrombectomy approach, patients with mild stroke and LVO at presentation or patients with infarct size beyond 70 mL and LVO who may benefit from mechanical thrombectomy. In addition, there is a current paucity of data to support treatment of patients with LVO in the posterior circulation, requiring future research including randomized clinical trials.

## 5. Conclusions

The field of acute stroke treatment has made tremendous progress in reducing the overall burden of disability. Our understanding of the pathophysiology of acute ischemic injury, neuroimaging to quantify the extent of penumbra and infarction, and acute stroke reperfusion therapies have contributed to these advancements. Ongoing research will help to refine treatment protocols and offer treatment to all AIS patients who can benefit.

## Figures and Tables

**Table 1:** Current Inclusion and Exclusion Criteria for IV alteplase based on AHA/ASA 2019 guidelines.

**Inclusion Criteria**
Age ≥ 18 years
Persistent disabling neurological deficits suggestive of an acute ischemic stroke
Onset of symptoms between 3 to 4.5 h from LKW (Level I) or Wake-up and unknown time of onset of symptoms, based on DWI-FLAIR imaging mismatch (level II)
**Exclusion Criteria**
Significant head trauma or prior stroke in the previous 3 months
Symptoms suggestive of SAH
History of previous ICH
Intraaxial-Intracranial neoplasm
Recent intracranial or intraspinal surgery within prior 3 months
Gastrointestinal malignancy or hemorrhage within 21 days
Known aortic arch dissection or infective endocarditis
Elevated blood pressure (systolic > 185 mm Hg or diastolic > 110 mm Hg)
Active internal bleeding in the past 21 days
Acute bleeding diathesis, including but not limited to:
Platelet count < 100,000/mm^3^
Therapeutic doses of low molecular weight heparin received within 24 h (e.g., to treat VTE and ACS); this exclusion does not apply to prophylactic doses (e.g., to prevent VTE)
Current use of anticoagulant with INR > 1.7 or PT > 15 s
Current use (i.e., last dose within 48 h in a patient with normal renal function) of a direct thrombin inhibitor or direct factor Xa inhibitor with evidence of anticoagulant effect by laboratory tests such as aPTT, INR, ECT, TT, or appropriate factor Xa activity assays
Blood glucose concentration < 50 mg/dL (2.7 mmol/L) unless corrected prior to administration
CT Head demonstrating multilobar infarction or hypodensity involving >1/3 cerebral hemisphere
Extensive regions of obvious hypodensity consistent with irreversible injury
**Careful considerations in which IV alteplase can be considered (weighing risks and benefits)**
Arterial puncture at noncompressible site in previous 7 days
Symptoms most consistent with a stroke mimic
Seizure at onset with postictal residual neurological impairments
Only minor or rapidly improving stroke symptoms
Pregnancy
Known systemic malignancy with reasonable life expectancy and no known coagulopathy
Pre-existing disability with mRS of ≥2
Major surgery or serious trauma (excluding head trauma) within previous 14 days
Previous gastrointestinal or urinary tract hemorrhage (beyond 21 days)
Recent acute myocardial infarction (within previous 3 months)
Untreated intracranial vascular malformation
Large (≥10 mm), untreated, unruptured intracranial aneurysm

Abbreviations: LKW, last known well; DWI, diffusion-weighted imaging; FLAIR, fluid attenuated inversion recovery; SAH, subarachnoid hemorrhage; ICH, intracranial hemorrhage; AVM, arteriovenous malformation; GI, gastrointestinal; aPTT, partial thromboplastin time; INR, international normalized ratio; IV, intravenous; PT, prothrombin time; mRS, modified Rankin scale.

**Table 2:** Summary of the Main Trials Evaluating Mechanical Thrombectomy with Retrievable Stents for Acute Ischemic Stroke.

	MR CLEAN	ESCAPE	EXTEND IA	SWIFT PRIME	REVASCAT	THRACE
Patients number (treatment vs. control)	500 (233 vs. 267)	315 (165 vs. 150)	70 (35 vs. 35)	195 (98 vs. 98)	206 (103 vs. 103)	414 (204 vs. 208)
Median age in years (treatment vs. control)	65.8 vs. 65.7	71 vs. 70	68.6 vs. 70.2	65 vs. 66	65.7 vs. 67.2	66 vs. 68
LKW to randomization	6	12 h, image to puncture <60 min	6	6 h, image to puncture <90 min	3.7 h	4.5 h, onset to groin puncture 5 h
Inclusion criteria	Age ≥ 18, NIHSS ≥ 2	Age ≥ 18, any NIHSS (disabling) Barthel index > 90	Age ≥ 18, any NIHSS, premorbid mRS < 2	Age 18–80 NIHSS ≥ 8 Premorbid mRS < 2	Age 18–80 NIHSS ≥ 6 Premorbid mRS < 2	Age 18–80 NIHSS 10–25
Imaging	CTA +/− CTP Any ASPECT	CT ASPECTS 6–10 with good collaterals >50% of MCA	CTA/CTP (core < 70 mL)	CTA +/−CTP ASPECT 6–10 No cervical ICA occlusion	CTA ASPECT 7–10	CTA or MRA Any ASPECT
NIHSS (treatment vs. control)	17 vs. 18	16 vs. 17	17 vs. 13	17 vs. 17	17 vs. 17	18 vs. 17
ASPECT (treatment vs. control)	9 vs. 9	9 vs. 9	Not reported	9 vs. 9	7 vs. 8	Median not reported
TPA (treatment vs. control)	87.1 vs. 90.6	72.7 vs. 78.7	100 vs. 100	100 vs. 100	68.0 vs. 77.7	100 vs. 100
Median onset to groin puncture in min	260	185	210	224	269	250
Onset to reperfusion in min	Not reported	241	248	250	355	303
M1 occlusion	66.1 vs. 62	68.1 vs. 71.4	57 vs. 51	67 vs. 77	64.7 vs. 64.4	86 vs. 79
TICI score 2b-3%	58.7	72.4	86	88	65.7	69
MRS 0–2 at 90 days %	32.6 vs. 19.1 OR 1.7 (95% CI 1.2–2.3)	53 vs. 29.3 OR 1.8 (95% CI 1.4 2.4)	71 vs. 40 OR 4.2 (95% CI 1.4–12)	60.2 vs. 35.5 OR 1.7 (95% CI 1.2–2.3)	43.7 vs. 28.2 OR 2.1 (95% CI 1.1–4.0)	53 vs. 42 OR 1.55 (95% CI 1.05–2.30)
MRS 0–2 at 90 days NNT	7.1	4.2	3.2	4	6.3	9.1
sICH%	6 vs. 5.2	3.6 vs. 2.7	1 vs. 3	1 vs. 3	1.9 vs. 1.9	2 vs. 2

Abbreviations: LKW, last known well.

## Data Availability

Not applicable.
